# The lived experiences of fatigue among patients receiving haemodialysis in Oman: a qualitative exploration

**DOI:** 10.1186/s12882-024-03647-2

**Published:** 2024-07-29

**Authors:** Zakariya Al-Naamani, Kevin Gormley, Helen Noble, Olinda Santin, Omar Al Omari, Huda Al-Noumani, Norah Madkhali

**Affiliations:** 1 Lecturer in Nephrology and Palliative Support Armed Forces Medical Services School, Ministry of Defense, Al-Khoudh, Postal Code 111, Muscat, P.O .Box 721, Oman; 2 School of Nursing and Midwifery, Queen’s University Medical Biology Centre, Belfast, BT9 7BL UK; 3 College of Nursing and Midwifery, Mohammed Bin Rashid University Of Medicine and Health Sciences, Dubai, United Arab Emirates; 4 Mental Health College of Nursing, Sultan Qaboos University, Al-Khoudh, Postal Code 123, P.O.Box 66, Muscat, Oman; 5 Cancer Nursing and Palliative Care, College of Nursing, Jazan University, Jazan, Saudi Arabia

**Keywords:** Fatigue and contributing factors, Perspective, Omani patient, End-stage kidney disease, Haemodialysis, Sexuality.

## Abstract

**Background:**

Fatigue among patients with end-stage kidney disease (ESKD) receiving haemodialysis imposes a substantial burden on patients’ quality of life and expected treatment outcomes. This study explores the perspective on ESKD-related fatigue and contributing factors among Omani patients receiving haemodialysis.

**Methods:**

An exploratory qualitative design was used. Participants (*N* = 25) were recruited from two Omani haemodialysis centres, and data were collected through semi-structured interviews, which were transcribed and analysed using a thematic analysis approach. NVivo 11 is used to manage qualitative data and create memos, nodes, and codes.

**Results:**

Findings highlighted three themes: (i)“Inevitability of fatigue,” (ii)“Contributors to physical fatigue,” and (iii)“Contributors to mental fatigue.” Theme one alluded to the inevitability of fatigue and the unique experience encountered by patients. Theme two addressed the physical fatigue associated with ESKD-related factors, such as chronically low haemoglobin levels, and the exhausting impact caused by the frequency and travelling distance for treatment sessions. Theme three, mental fatigue, was notably driven by heightened emotional disturbance, encompassing frustration, guilt, anxiety, and distress, that in turn impacted family interactions, frequently triggering anger and remorse. Moreover, mental fatigue is a result of disturbances in expressing physical sexuality in marriage, as physical fatigue was found to be a significant contributor to unsatisfactory sexual experiences and, thus, straining the relationships between couples.

**Conclusions:**

This study offers an explanation of fatigue among Omani patients with ESKD who are receiving haemodialysis. The study emphasises close links between physiological change, the haemodialysis process, and mental tiredness, together with their contribution to supporting the need for a holistic approach and care strategies in managing these patients and promoting patient and family well-being.

## Background

End-stage kidney disease (ESKD) imposes a substantial burden on global healthcare services and patients’ quality of life. According to the National Institute of Health 2022 annual report, the prevalence of ESKD increased by 118.7% between 2000 and 2019 [1]. Even higher percentage was reported in Arabic countries, including Oman, where a significant increase was mainly attributed to diabetes and hypertension as the primary causes of ESKD [1]. In Oman, statistics highlight the accelerating incidence of ESKD and the consequent need for renal replacement therapy (RRT), which stood at 21 per million people (pmp) in 1983 compared with 160 pmp in 2019 [2]. In 2019, a total of 4117 patients with ESKD received RRT compared to 49 patients in 1983 [2].

Globally, haemodialysis is the most common RRT. Although haemodialysis plays an essential role in sustaining the life of patients with ESKD, patients often experience debilitating symptoms that negatively affect their physical, psychological, and social well-being [3, 4]. Flythe et al. (2018) listed the key symptoms presenting among patients receiving haemodialysis, including fatigue, muscle cramping, body aches, insomnia, anxiety, depression, and frustration [5]. Despite the advancement in haemodialysis technology and delivery care, poor symptom management, such as those identified, remains a health professional concern [5–7].

Fatigue is one of the most reported symptoms among haemodialysis patients, with a prevalence rate ranging from 49 to 92% [8–10]. Fatigue, characterised by an overarching sense of physical and mental exhaustion, significantly impairs patients’ ability to engage in daily activities, maintain social connections, and experience a satisfactory quality of life [11, 12]. Fatigue was found to be correlated with increased risks of cardiovascular disorders [13], suicidal attempts [14], mortality rate [15], and adherence to the haemodialysis regimen. Also, it reduced the likelihood of kidney transplantation [10]. Despite the significant negative impact of fatigue among patients receiving haemodialysis, fatigue-causing mechanisms, and its contributing factors are still unclear and poorly investigated, leading to poor management strategies and a negative influence on patients’ quality of life and potential treatment outcomes [8, 16].

Fatigue among patients receiving haemodialysis is a complex phenomenon associated with multiple contributing factors resulting from the actual kidney disease process in the first instance and then the chronic haemodialysis regimen [16–19]. Fatigue is associated with multiple physiological [19], psychological [20, 21], and social [3, 22] contributing factors. For example, fatigue manifestations are attributed to poor sleep quality [23], family and social responsibilities [12], and mood disorders [21, 24], and the causes of this symptom are often ambiguous and confounded by cultural differences [22, 25, 26].

A number of studies have explored fatigue among patients receiving haemodialysis within European and North American contexts where culture and beliefs are likely to influence patient perspectives and interpretation of symptoms and will be uniquely different to other areas of the world [3, 27]. There is a dearth of studies in the Arab context exploring the lived experiences of fatigue among patients receiving haemodialysis. Oman is located in the southeastern corner of the Arabian Peninsula, with a land area of around 309,500 km [28]. The majority of Omanis are Arabs and Muslims who are proud of their heritage and culture. According to the 2022 annual health report of the Ministry of Health, the total population in Oman is approximately 4.9 million, comprising Omanis (59%) and non-Omanis (41%). The Omani population is relatively young, with 94% below the age of 60 [28]. Addressing this gap is important because of the unique cultural, environmental, and lifestyle factors related to the Arab context. This study aims to explore the experience of fatigue and contributing factors among Omani patients receiving haemodialysis.

## Methods

### Study design

An exploratory qualitative research design was used to gain in-depth understanding of the experience of fatigue and contributing factors among patients with ESKD receiving haemodialysis. The exploratory qualitative approach is used to gain new, rich insights and understanding of the various ways in which a phenomenon is manifested and the underlying process that underpins it as presented by participants [29]. This is particularly important where the topic of investigation or phenomenon is not fully understood, and there is limited coverage in the literature about the experience of fatigue among Omani patients receiving haemodialysis [29].

### Study participants

The study participants were recruited from two in-centre haemodialysis in Oman. The centres run four haemodialysis sessions daily: a morning session (7 am – 11 am), an early afternoon session (12 noon – 4 pm), a late afternoon session (5 pm – 9 pm), and a night-time session (10 pm – 2 am). Participants are required to attend three in-centre haemodialysis sessions a week for four hours per session. The scheduled sessions are fixed each week for patients by the healthcare provider according to the slot availability unless the change is necessary.

Eligibility criteria included adult Omani patients over 18 years old, diagnosed with ESKD, recipients of haemodialysis for at least six months, and reporting a score of ≥ 1 of fatigue using a Likert scale from 0; no fatigue to 10; severe fatigue. A period of 6 months on haemodialysis was deemed sufficient time for patients to adapt to and cope with the treatment process of haemodialysis in their daily lives. This time window was selected because, in the first period of haemodialysis, patients experience a critical transition in their life and have significant physiological and functional changes that potentially affect how they perceive their fatigue experience [30]. Participants were excluded if they had a cancer diagnosis, HIV, severe heart failure, or depression, as these patients were likely to experience fatigue unrelated to haemodialysis.

### Data collection

After ethical approval, an Interview Guide was formulated and piloted in a different location with three patients receiving in-centre haemodialysis to ensure the questions were clear and relevant to haemodialysis-related fatigue (Table 1). Price (2002) emphasised using a framework and a sequential questioning system that begins with the least intrusive and moves on to more in-depth concepts if the interviewee indicates their readiness [31]. A minor modification related to sexual effects in the guide was included to address cultural aspects. These patients and their interviews were excluded from the final analysis.

A flyer was posted on the notice board, and interested potential participants contacted the senior nurse, who has a copy of the information sheet, contact consent form, and knowledge about the study and researchers. The information sheet stresses that this study is independent from the hospital and that declining participation will not affect the patients by any means. If patients were willing to participate, they were asked to fill out a contact consent form and return it inside envelopes to the haemodialysis centre reception desk at their next appointment. Participants were also given the option to contact the researcher directly via phone, as provided on the information sheet, for any clarifications. After collecting the contact consent forms, the researcher (ZA) called participants and provided a detailed explanation about the study. Of 76 potential participants approached to participate, 30 participants agreed to take part in the semi-structured interview. Participants were then contacted by the main researcher (ZA), who met with them in a mutually accepted place and time.

After obtaining formal consent, the interview process began with an informal discussion to establish rapport, create trust, and facilitate flexibility in the interview [32]. After that, the researcher started first with the general and the least personal questions to break the ice [31]. Then, the researcher directed the interview and asked more personal and probing questions to elicit detailed information, which ultimately advanced the research goals.

After 25 interviews, no additional information was found, and data saturation occurred [33]. Therefore, the remaining five interviews were cancelled. All interviews were held in a convenient, quiet room for 30 to 80 min. The interviews were recorded and transcribed verbatim, and field notes were taken during interviews. Data were stored in a protected and encrypted computer to ensure data security and protect participants’ privacy.

**Table 1:** Framework for semi-structured interview questions

Type of question		Questions	Probing Questions
Warm-up		Could you introduce yourself? Could you tell me about the symptoms that you have experienced since you started haemodialysis?	What is your name, marital status, age, number of children, area you work in, education, how long you have been receiving haemodialysis?
Experience	Feelings	Can you tell me about the fatigue you have experienced since you started haemodialysis?	When you hear the word fatigue, what does it mean to you? How does it feel? How would you describe it?
	Attitudes/feelings and actions	How does fatigue impact your life and daily activities? (Physical, psychological, social and sexual)	How does your fatigue affect in your life? Can you provide examples of how fatigue affects your daily activities?

### Ethical approval

The ethical approval was obtained from the ethical committee of the Ministry of Health in Oman (MOH/CSR/18/9002). This study is consistent with the Helsinki Declaration. Participants were informed that they could refuse to answer questions or withdraw at any time. A protocol was developed to protect interviewees from potentially provoking emotional distress during the interview. ZA, who conducted the interview, is a senior nurse with a lot of experience and was able to survey the participants for any sign of stress during the interview. If any sign of stress was shown, the interview was stopped till the participant relaxed and their ability to continue the interview was assessed, but if the participant continued showing the signs of stress, the interview was terminated, and the participants would be referred to a psychologist who was attached to the unit. All Omanis are covered by health insurance so that no expenses would be paid. However, the researchers did not experience this in any case. Pseudonyms were used instead of identifying information to maintain participants’ confidentiality.

### Data analysis

Semi-structured transcripts were analysed systematically via using the six-step approach to thematic analysis, namely (1) familiarisation with the data (reading and re-reading), (2) generating initial codes, (3) searching for themes, (4) reviewing themes, (5) defining and naming themes, (6) producing the results [34].

At the initial stage of data management, all interviews were transcribed verbatim, with interviews conducted in Arabic. All the interviews were translated into English. WHO guidelines for translation were followed [35]. First, translation to English was conducted by a researcher (ZA), who is fluent in both languages, and back translation was conducted by another fluent researcher (OO) to ensure the data did not lose their richness. ZA). Then, the translated interviews were thoroughly checked for accuracy by other researchers fluent in Arabic and English to validate the translated information (HS & NM). NVivo 11 was used to manage the raw data, help organise the dataset, and create memos, free nodes, and tree nodes to show the relationship between the codes and themes.

**Fig. 1:**
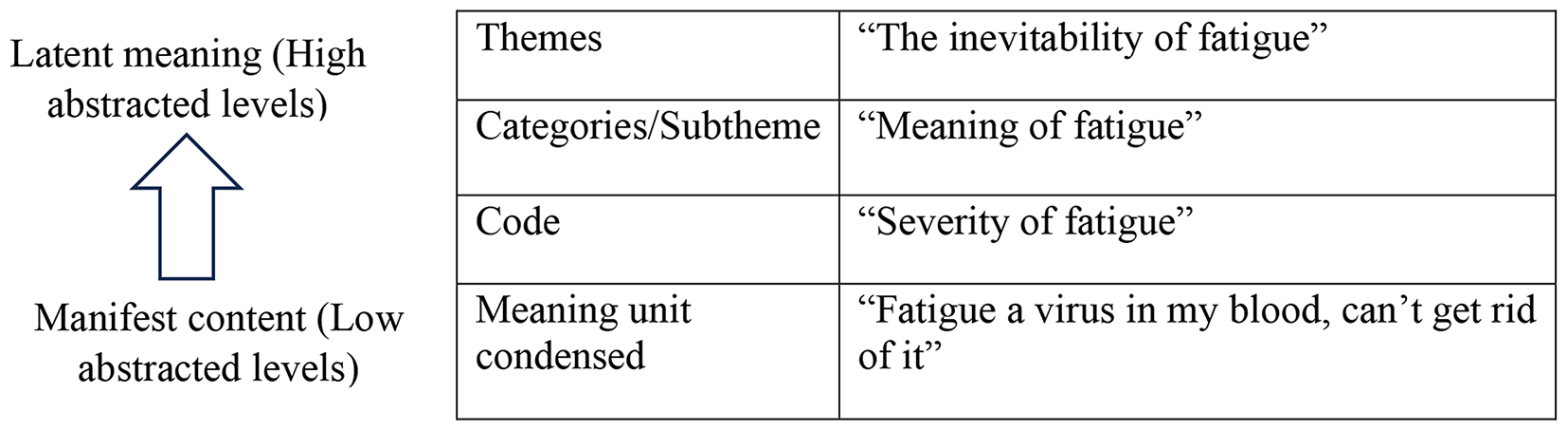
Example of the data reduction process

Descriptive encoding entailed reading the transcripts several times to create accurate representations of the raw data and to get a general sense of the key concepts in the descriptions [36]. The text was then divided into manageable meaning units. The meaning of the text was preserved even after these meaning units were further reduced. These condensed units had codes assigned to them. Codes that were comparable and related were categorised. The researcher performed additional analysis in each category to generate overarching themes, interpretive themes, and subthemes (Fig. 1). As a quality measure, an expert panel (KG, HN, and OS) conducted a comprehensive review of the findings to validate the authenticity and precision of the interpretations [37].

Miles and Huberman Framework (1994) was used to ensure a systematic, rigorous and transparent process of data analysis and interpretation [38]. The framework includes three main processes: data reduction, data display and drawing conclusions. Data reduction was achieved by researchers (ZA, KG, HN, OS) who independently reviewed data to capture the essence of the data and identify initial emerging codes. The initial codes were then displayed and organised to identify patterns and themes, and discrepancies were resolved by consensus and mutual understanding. Researchers then engaged in iterative processes of discussions to draw conclusions and verify their interpretations by moving back and forth between the themes and text to ensure the validity and reliability of the findings.

To further ensure the quality of the data and the rigour of this qualitative study and maintain the trustworthiness, the researcher adhered to the four criteria Lincoln and Guba proposed: credibility, transferability, confirmability, and dependability throughout the study [36]. These criteria are considered important elements in qualitative research to ensure the accuracy of the process of data interpretation and how conclusions are drawn. Several methods were used; for example, all interviews were digitally audio recorded and transcribed verbatim to ensure no information was lost and checked independently (credibility and transferability). Pseudonyms ensured participants’ confidentiality and anonymity of information, and direct quotes from the interviews were provided to authenticate the meanings conveyed through various themes (transferability). Interpretations of the findings were further verified for accuracy and completeness by expert researchers (confirmability).

## Results

Table 2 outlines the patients’ sociodemographic profiles. The mean age of patients was 41.32 years. The length of time they had been receiving haemodialysis treatment ranged from 1 to 17 years. Most patients were married (68%) and employed (44%).

**Table 2:** Patients’ sociodemographic data

Ethnicity		*N*
Omani		25 (100%)
Age		Mean (range)
Total sample (*n* = 25)		41.32 (23–62)
Female (*n* = 9)		41.37 (23–49)
Male (*n* = 16)		41.30 (27–62)
**Education**		**N**
Bachelor degree		7 (28%)
Diploma degree		6 (24%)
School degree		12 (48%)
**Marital status**		**N**
Single		7 (28%)
Married	Average No. of children = 3.70 (0–9)	17 (68%)
Divorced		1 (4%)
**Employment status**		**N**
Full time		11 (44%)
Retired		7 (28%)
Unemployed		7 (28%)
**Living arrangement**		**N**
With own family		16 (64%)
With Parents		6 (24%)
With Husband’s family		2 (8%)
With son		1 (4%)
**Dialysis duration/ yrs**		**Mean (range)**
Total sample (*n* = 25)		5.52 (1–17)
**Timing of dialysis sessions**		**N**
Morning		6 (24%)
Early afternoon		8 (32%)
Late afternoon		5 (20%)
Night		6 (24%)

The qualitative thematic analysis revealed an overarching theme, **Inevitable and Multifaceted: ESKD-related fatigue**, highlights patients’ perspectives on the meaning and description of fatigue and considers some of the factors which impact on fatigue severity. Generally, patients found it difficult to clearly describe their fatigue because its contributing factors were unclear.

What to say about my fatigue? It’s difficult to explain the feelings … (BHC: P23).

This overarching theme is further divided into three interpretative themes: (1) inevitability of fatigue, (2) contributors to physical fatigue, and (3) contributors to mental fatigue (Table 3). Although the factors contributing to physical and mental fatigue are discussed individually, these factors are strongly interrelated and as such has an impact on one another.

**Table 3:** List of emergent themes and subthemes

Overarching theme	Interpretative theme/Categories	Subthemes
Inevitable and Multifaceted: ESKD-related Fatigue	1. Inevitability of fatigue	b. Meaning of fatigue c. Nature of fatigue
	2. Contributors to physical fatigue	a. Negative physiological changes b. Nature of haemodialysis procedure c. Night-timing of haemodialysis sessions
	3. Contributors to mental fatigue	a. Heightened emotional disturbances b. Disturbance in sexuality and marriage

### Inevitability of fatigue

Most patients perceived fatigue as an inevitable experience and to be expected as a consequence of haemodialysis. Insights around this theme were articulated through patients’ individual descriptions of what fatigue meant for them, and their attempts to describe the fluctuation in the severity of their fatigue.

### Meaning of fatigue

Patients believed fatigue to be a symptom closely related to their disease and treatment and, therefore, to be expected and unavoidable. Fatigue was described as a constant presence that followed patients continually, and was described as being like a ‘virus’ in the bloodstream. They described the impact of fatigue on their physical and mental well-being and highlighted how fatigue negatively impacted their everyday lives.

*Doing haemodialysis is exhausting physically and mentally because my life changed totally … This fatigue is like a virus in my blood, I can’t get rid of it.* (BHC: P20)

Some patients believed that their fatigue was a consequence of abnormal physiological values arising from their disease, particularly chronically low haemoglobin levels. Alongside this, the unavoidable weight gain caused by accumulated body fluid, was also felt to have a negatively impact on their well-being.

*You know our haemoglobin now is below the normal level. … So, fatigue is always present but sometimes I feel less tired, and this is normal because since I started my haemodialysis treatment, my haemoglobin has been between 7 and 9 [g/dl].* (BHC: P09)

Other patients believed that physical fatigue was an inevitable consequence of the constant and exhausting treatment process and the frequent travel for these treatments, in some cases this was up to three times per week.

*After my dialysis session I feel exhausted until the next day* (BHC: P13).

The physiological changes associated with haemodialysis increased the severity of physical fatigue and contributed to patients’ inability to meet their daily responsibilities. Patients often complained of feeling overwhelmed when they thought about their roles and responsibilities at home; and mentally tired and challenged.

*Even if my physical fatigue is less, I feel mentally tired, which sometimes gives me a headache … because I’m still young and [I] worry for my future. … [M]y family also suffer with my treatment and the mood fluctuations that I’ve suffered from since I started haemodialysis.* (BHC: P08)

### Nature of fatigue

Patients reported that the severity of their fatigue was unpredictable and often fluctuated throughout the day, thereby negatively affecting their capacity to meet their daily needs. Most of the patients stated that whenever haemodialysis treatment was completed, they frequently complained about post-dialysis fatigue (PDF), which many of them seemed to accept as an inevitable experience, and which was so severe that it often limited their functional abilities. PDF is immediately manifested after haemodialysis sessions and may persist for hours due to fluid shifts during the cycle of haemodialysis.

*I feel exhausted when I arrive home … I feel remorse at not being able to do household tasks and take care of my husband and children.* (BHC: P25)

Fatigue and fatigue severity following haemodialysis was described as unpredictable with various recovery times. Patients reported that sometimes they would experience minimal PDF, and hence required only a little rest before they were ready to engage in other activities. On the other hand, there were times when the fatigue persisted for longer than a full day, as this patient explained:

*Fatigue varies day to day. Sometimes, I feel fatigue for 24 h, and at other times, I just feel fatigued for 4 h, but I’m not [always] sure if I can do work.* (Phase 1: NHC: P05)

These fluctuations in the severity of fatigue meant that patients described their fatigue experience in different ways. When their fatigue was less severe, especially during haemodialysis sessions, they used terms such as ‘tired’ or ‘weak’ to describe their feeling of fatigue. However, when the symptom of fatigue was severe, they described the fatigue phenomenon using a range of descriptive words such as ‘paralysing’, ‘lethargic’ and ‘exhausting’. This distinction was underscored by their body language when they narrated their severe fatigue experience. They looked sad and despondent when describing severe fatigue, and conceptualised it as an unavoidable feeling that they were left to face alone, because healthcare providers (HCPs) rarely discussed fatigue with them after haemodialysis sessions,

*I feel my whole body is paralysed and exhausted, like I’ve a mountain on my back and pain in my joints. I can’t walk or do anything* (pauses with a blank expression). *The feeling can’t be described …* (spoken with a sad tone) *… Nurses never ask about [my] fatigue, [except] only when I experienced recurrent low blood pressure during haemodialysis ….* (NHC: P02)

Some patients described themselves as ‘fatigued persons’, having surrendered or accepted fatigue as an inevitable part of their haemodialysis day. Sadly, due to severe fatigue, some patients had become accustomed to simply excluding the haemodialysis day from their planning for the week, knowing full well that these days would be “wasted” anyway.

*Fatigue is always present, especially after my dialysis sessions … That is why I don’t count the dialysis day as part of my week.* (BHC: P13)

### Contributors to physical fatigue

#### Negative physiological changes

Patients were aware that physiological changes contributed to their physical fatigue. Fatigue was viewed as a consequence of electrolyte disturbance, especially potassium level.

*When my potassium level is high, my heart rate increases, and I can feel it. I feel very tired and I’m afraid my heart will stop …* (BHC: P25).

High levels of phosphorus in the blood caused continued and intolerable itching, especially in summer with heightened temperature. Patients associated symptoms of irritation, pain and physical discomfort to their feeling of fatigue, particularly when performing daily activities. Some patients also complained of sudden muscle cramps, especially at night. They highlighted as negatively impacting sleep quality, often leaving them further exhausted the next day, as these participants narrated:

*If I’m exposed to sunlight especially during summer, I get itching and sometimes muscle cramps. I can’t sleep peacefully at night because of scratching or sudden muscle cramps that cause severe pain* (shows grimaced face). *Next morning, I feel very tired when doing my work* … (NHC: P04).

A number of patients also linked their energy levels to their haemoglobin levels, they believed that their energy levels dropped in parallel with their haemoglobin levels.

*If my haemoglobin level drops below 8, I can’t do my work, even simple tasks. I feel lethargic, sleepy, and I just stay at home …* (BHC: P01).

The issue was of particular concern for female patients, who indicated that their menstrual periods caused a further drop in their haemoglobin levels, resulting in further increase in the severity of their fatigue:

*I think the cause of my fatigue is low haemoglobin level … when I get bleeding, it [my haemoglobin] drops to 5 and I feel more tired and cannot do anything at all, and you know this thing is not in our hands as we get it [menstrual period] monthly.* (BHC: P07)

Furthermore, some patients linked increased physical fatigue levels to the extra fluid that accumulates between haemodialysis sessions. This association between fatigue and weight gain became apparent, especially on Fridays when dialysis centres were officially closed for the weekend. This means that patients often have to endure two consecutive days without haemodialysis. During this time, there was also a tendency to consume more fluids because of social activities and family gatherings, which exacerbated weight gain and further increased their physical fatigue.

*You know, during the weekend, I eat and drink more because of family gatherings and activities we have. Sometimes I gain more weight, approximately 5 to 6 kg. This really makes me feel more tired.* (BHC: P05)

#### Nature of haemodialysis procedure

Many patients observed that when they attended haemodialysis sessions, the healthcare providers’ efforts to remove accumulated fluid often aggravated their post-dialysis fatigue and increased their risk of developing side effects such as hypotension and electrolyte imbalances, which in turn further extended their recovery periods. This feeling of immense fatigue was especially present when the healthcare providers attempted to remove more fluid than the patients could otherwise tolerate,

*[I]f they [HCPs] remove more fluid than my body can tolerate, my blood pressure drops and I feel nauseated, drowsy and very weak*. (NHC: P01)

Some patients also believed that the pump (dialysis flow rate) used to maintain circulating blood flow while removing accumulated body fluid was sometimes set at an intolerably high level (of up to 400 ml/min).

*… the nurse used to keep the pump speed at 400 [ml/min], and I lost consciousness many times and at home I was very exhausted… till they adjusted it to 300 and I feel somewhat better.* (BHC: P13)

In addition to fatigue, patients experienced other recurrent unpleasant symptoms during the haemodialysis sessions, the most common being hypotension. These unpleasant symptoms, when they occur, aggravate the severity of fatigue and the recovery time.

*[I]f they continually kept the speed on the higher side for a long period of time, I could develop heart problems later on as my blood vessels would become weaker, and I would be more at risk of [developing] hypotension …* (BHC: P23).

#### Night-time haemodialysis sessions

Unfavorable timing of haemodialysis sessions, particularly at night (10 pm – 2 am) was offered as a reason for additional or exacerbated fatigue. This caused further concern to some patients who commented that night-time haemodialysis sessions resulted in even more debilitating fatigue due to what they argued as a combination of post-dialysis fatigue and insufficient night sleep. This in turn was further worsened by the need of some patients to drive back home by themselves,

*… I stop at least twice to rest and take a nap because I feel very tired and sleepy, especially during night sessions … It’s not good to drive in this condition but I don’t have a choice.* (BHC: P08)

Many patients reported that the severe fatigue following night-time haemodialysis sessions predisposing them to road traffic accidents.

*I felt more exhausted when I did night sessions because I was not getting enough sleep at night, and I had to drive home at 3:30 am… I fell asleep twice while driving and had an accident.* (NHC: P05)

#### Contributors to mental fatigue

##### Heightened emotional disturbances

Many patients said the burden of fatigue was provoked by a variety of emotions, including frustration, guilt, anxiety, and distress. Many felt they could neither spend enough quality time with their families nor fulfill their needs due to the unpredictable nature and severity of fatigue.

*I feel psychologically exhausted, as I can’t handle my fatigue and home responsibilities as a mother, … I feel guilty if they come back and their lunch is not ready, …* (BHC: P25).

Some reported that post-dialysis fatigue increased their frustration and anger, especially when subjected to noise, or when they were too exhausted to function after haemodialysis sessions. Often, frustration impacted negatively on their behaviour and resulted in inappropriate reactions, such as verbal abuse toward others. Patients felt judged by these emotional outbursts and were sometimes criticised for being angry unnecessarily. Participants reported that their emotions disrupted them psychologically, and they often felt huge remorse for angry outbursts, especially towards close family members,

*“After dialysis sessions I can’t control my temper. Imagine, I become annoyed with simple things, and I react in a bad way, like throwing things. …* (BHC: P13).

*Sometimes, I shouted, and no one could tolerate my temper. Believe me, I used to be calm and rarely got angry but now even my husband has told me my behaviour has changed …* (BHC: P18)”.

Anxiety was frequently experienced by the patients in this study. Causes were related to a lack of consistency in the haemodialysis services as well as a lack of essential clinical staff and other resources. This caused overwhelming thoughts as they were unsure about the quality of care provided and the impact on fatigue management.

*She [the dietician] comes only during morning and early afternoon sessions, and she sees only patients who have abnormal blood results. There is no dietician in the remaining sessions and there were fewer services when I did late evening sessions.* (BHC: P18)

Patients experienced a sense of isolation, loneliness, and worry associated with the inability to be involved in social activities. Most patients often felt socially isolated. They explained that post-dialysis fatigue reduced their interest in social interaction and demotivated them because they were uncertain of their moods and capabilities. They were ashamed to be seen as ‘a weak person’ and unable to spend enough quality of time with their families and friends

*I seldom join my family or friends for trips or special occasions, and I prefer to stay home because I’m not sure about my energy, which might ruin the trip. … I also used to go out for shopping with my sisters and sometimes I felt exhausted after the shopping. Therefore, I don’t feel interested in going out anymore.* (BHC: P15)

Financial insecurity, especially among those who were employed, also caused huge concern. The most crucial source of worry was the possibility of losing a job or missing out on promotion, thus affecting future income.

*… When I started haemodialysis, and because I can’t function as before, the company forced me to retire from work. It is not easy to survive, and I keep thinking how I can provide a better life for my family* (sad expression). (NHC: P06)

##### Disturbances in sexuality and marriage

Fatigue led to a reduction in physical sexual contact between partners. Many patients had lost interest in intimacy and the desire to engage in sexual relations with their partners, which they argued was a consequence of continued fatigue. Some reported that they engaged in sexual intercourse just to satisfy their partners. This poor sexual experience negatively affected some relationships. Patients worried about the feelings of their partners, whether expressed or not, and were aware that unsatisfactory sexual relationships were “hot spots” in influencing their married life.

. *...Sometimes, I can’t tolerate it [Sexual relationship] and don’t finish because I feel exhausted and have no stamina. I sometimes fake my feelings during the relationship to satisfy my wife* (said with sad expression). (NHC: P06)

In addition to physical fatigue, poor sexual performance in males was also attributed to erectile dysfunction, which often caused severe anguish, with many suffering in silence about their sexual inability. However, while many suffered in silence, others sought alternatives to aid their sexual performance, as this participant explained:

*I get weak erections, and sometimes I use Viagra tablets to enjoy [the sexual relationship] more.* (NHC: P06)

Patients were also concerned about their weight gain and fistulas, which they perceived as making them less attractive to their partners. They also expressed concern about damaging the fistula site during sexual intercourse, concerns which they reported their partners also shared,

*My performance during [sexual] relations became less [frequent]. I feel heavy and [I] become tired … we [the participant and her husband] are also too concerned about the fistula, we might unintentionally damage it … during that moment [of sexual intimacy].* (BHC: P18)

Unmarried patients expressed anxiety about finding a husband or wife. Their greatest worry was whether they would ever find someone who would accept their current situation, including their limitations. Some felt hopeless about the possibility of having a life partner:

*I don’t know if I will be able to get married. Kidney failure and dialysis treatment is like an imprint on my life. Honestly, I’m losing hope in getting a wife because no one will accept a person [who is] sick and retired from work.* (BHC: P08)

Patients who experienced previous rejections lamented that they were left psychologically broken, unsure if they should continue to seek a spouse.

. *I’m turning 43 years [old]. I’m still not married, and it’s really difficult not being able to fulfil my life and have my own family.* (BHC: P24)

Some married younger adults expressed worries regarding their fertility and the possibility of failing to have children of their own.

*… My husband suffers sometimes, and I feel guilty because we’ve no children yet. I know he wants to be a father but what I can do?* (Participant cries as she narrates her ordeal. (BHC: P20).

When asked how they dealt with difficulties related to sexual health, many patients reported being unsure about how to deal with such problems. Some were too shy and embarrassed to ask for professional support,

*I feel too shy and embarrassed to tell the doctor because it [sexual concerns] is a sensitive topic.* (BHC: P09)

## Discussion

Effective fatigue management requires a clear understanding of the symptoms experienced in relation to the affected person’s perception and evaluation of and response to fatigue. Notable findings in this study show that patients found it difficult to clearly describe their fatigue and its causes because of incidences of intrusive and often lingering physical and mental contributing factors. In fact, patients often evaluate what aggravates the severity of their physical and mental fatigue instead of its causes, which might link to the patient’s perception of the inevitability of fatigue. The finding that fatigue is present and persistent aligns with published literature [12, 39, 40]. For example, in a study conducted in the UK, haemodialysis patients reported that ‘fatigue is persisting despite rest’ [10]. Similarly, in Taiwan, patients described their experience as a ‘fight against fatigue all the time’ [41], while patients in Iran perceived fatigue as ‘a bad feeling’ that persisted, leading to endless physical and mental burnout [42]. In this study, too, haemodialysis patients often experienced ongoing physical and mental fatigue. Moreover, the severity of their fatigue symptoms worsened and became unpredictable after their haemodialysis sessions, leaving patients unable to organise or plan required daily activities [22, 42]. As a result, remarkable findings from this study where patients did not count dialysis days as part of their weekly plan due to unpredictable fatigue.

In terms of fatigue-causative factors, similar to other international findings [8, 22, 41], this study found that patients attributed physical fatigue to kidney disease and the exhausting haemodialysis treatment process. You et al. (2022) conducted a systematic review to investigate fatigue and its related factors among patients receiving haemodialysis. The authors found 26 potential risk factors associated with ESKD and the haemodialysis process, including electrolyte imbalance, low haemodialysis levels, and extra fluid that accumulates between haemodialysis sessions [8]. However, this study adds further perspectives about physical fatigue-related factors, including (1) female participants associated menstrual periods with the severity of fatigue, and (2) night-time haemodialysis sessions resulted in more debilitating fatigue due to insufficient night sleep. Therefore, expanding the bed capacity of the haemodialysis centres and eliminating night-time sessions has been recommended by patients to help mitigate their fatigue.

Mental fatigue was linked to role identity in specific cultural contexts, such as family gatherings and attending social events, as well as the specific gender roles in the family in fulfilling daily life. As observed in these studies, Lee et al. (2007) and Rezaie et al. (2020), for example, found that patients attributed emotional and psychological reactions to mental fatigue [41, 42]. Dealing with constant heightened emotions drained patients’ energy levels and negatively affected their behaviours and family relationships. Research also found that negative emotions such as lack of anger control, negative moods, and aggression were all associated with low tolerance thresholds that affect the severity of fatigue [3, 21]. Most patients, therefore, tend to exhibit some form of behaviour that indicates helplessness and the intention to avoid overwhelming emotions, such as avoiding social interaction, self-isolation, and limiting their social activities.

In this study, both male and female patients reported lowered sexual satisfaction with their spouse and reduced sexual activity and interest. All these factors lowered their self-esteem and increased their levels of frustration with themselves as well as their partner, leading to psychological distress. Jacobson et al. (2019) reported that haemodialysis patients lacked the stamina for sexual intimacy because fatigue inhibited sexual relations. Sexual activity drained their energy, which reduced their desire for sex, leading to a constant feeling of guilt over their failure to meet their partner’s sexual expectations. Female participants also tended to harbour the fear that their persistent fatigue would lead their partners to have extramarital affairs, predisposing them to mental distress [3].

The present findings move beyond these observations to offer insights from a transcultural diversity perspective. In Islam, major decisions are often patriarchal, which means that males have the authority to make decisions pertaining to divorce and marriage. Data revealed that the female participants were fearful that their spouse might leave them or take another wife because of their inability to satisfy their partner’s sexual needs, to conceive and give birth due to unceasing fatigue. Unmarried patients in the present study also reported mental fatigue arising from the fear of missing out on marriage and remaining single for life, of being rejected upon disclosing their illness, as well as feelings of unattractiveness following haemodialysis. This unmet need to get married had negatively affected patients’ identity and confidence. These findings concur with those of Stewart (2013), who argued that sexual concerns might negatively influence coping strategies among patients receiving haemodialysis. The author suggested that sexual problems could be further evaluated if one understood how patients perceived identity and potential family and social-cultural factors [43]. Gender and physiological differences between men and women could be explained by cultural and environmental factors which modify patients’ experience and interpretation of their fatigue. As a remedy, sexual education for haemodialysis patients and their partners is a vital aspect of managing sexually-related concerns [44]. Additionally, nephrology staff need an educational and training program to enhance their confidence in initiating discussions around sex-related problems among their patients [45].

Fatigue also had an impact on patients’ identity and self-esteem, especially when it came to expected gender roles. Gender roles are the socio-cultural expectations that encompass norms, behaviours, and actions of an individual based purely on them being male or female [46, 47]. In some cultures, gender roles and behaviours are well-defined: men are expected to take care of their families financially, whereas the primary role of women is to rear children and take care of their families [48]. In this present study, patients felt that fatigue rendered them incapable of fulfilling their gender role responsibilities. Similarly, Horigan et al. (2013), found that patients felt guilty because they were unable to meet their role responsibilities, had less confidence in their ability to care for their children, and felt themselves to be a burden on their family. The loss of their identity and self-esteem caused patients to experience additional psychological distress. Similar findings were reported in a systematic review [3] in which fatigue was found to erode patients’ identity and self-esteem.

Thus, the overlapping of these factors contributed to a state of confusion leading patients to describe their fatigue in relation to their physical, psychosocial, and behavioural problems. Identifying the causes of fatigue in such multi-dimensional environments, with social and cultural influences at play, can be challenging. It is therefore suggested that HCPs should independently assess the contributing factors of physical and mental fatigue to help patients gain a better understanding of fatigue and differentiate the various causative factors of their fatigue to manage it more effectively.

### Strength and limitation

The strengths of this study include a relatively large sample size and a well-defined population that provided valuable insights related to fatigue from their perspectives. There are also some limitations, including the timing of data collection; some patients were interviewed during dialysis sessions, which may have influenced the depth of the data obtained and may have hindered patients’ freedom to express themselves. Translation of some information from Arabic to English may have hindered data interpretation. Generalisation of the findings may be limited due to differences in the cultural background and haemodialysis services across countries.

### Implication for nursing education, practice, and research

Fatigue among patients receiving haemodialysis is a complex phenomenon and associated with physical and mental contributing factors. Thus, healthcare providers should assess and manage patients beyond traditional haemodialysis treatment and carry out regular holistic assessment to detect contributing factors impacting physical, psychosexual, and emotional fatigue. There is a need for formal and specific context-based guidelines to assess and manage fatigue within haemodialysis centres. Additionally, specialist educational and training programs for assessing and managing the complex symptoms associated with ESKD should be offered to HCPs. Further exploration of patient-initiated strategies to manage their fatigue is required, which may provide insights into potential fatigue-specific interventions.

## Conclusion

Fatigue perspectives among patients receiving haemodialysis are not well recognised [13] and obtaining in-depth knowledge of the complex nature of the multi-contributing factors of fatigue is essential. Understanding the meaning of fatigue and physical and psychological contributing factors may facilitate early assessment and better management. Therefore, healthcare providers need to be knowledgeable and aware of these factors to be able to recognise fatigue and offer appropriate care.

## Data Availability

The datasets used and analysed during the current study are available from the principal author upon reasonable request.

## Abbreviations

ESKD: End-stage kidney disease
RRT: Renal replacement therapy
PDF: Post dialysis fatigue
HIV: Human immunodeficiency virus
HCPS: Healthcare providers
